# The effect of vitamin D on morphine preference in rats: Possible biochemical and DRD2–GDNF signaling

**DOI:** 10.1002/brb3.2877

**Published:** 2023-01-11

**Authors:** Mahbubeh Saeedfar, Abolfazl Ardjmand, Behrang Alani, Amir Ghaderi, Hamid Reza Banafshe, Mohammad Esmaeil Shahaboddin, Gholamreza Ghavipanjeh

**Affiliations:** ^1^ Institute for Basic Sciences, Physiology Research Center Kashan University of Medical Sciences Kashan Iran; ^2^ Department of Physiology, School of Medicine Kashan University of Medical Sciences Kashan Iran; ^3^ Department of Applied Cell Sciences, Faculty of Medicine Kashan University of Medical Sciences Kashan Iran; ^4^ Department of Addiction Studies, School of Medical Kashan University of Medical Sciences Kashan Iran; ^5^ Department of Pharmacology, School of Medicine Kashan University of Medical Sciences Kashan Iran; ^6^ Research Center for Biochemistry and Nutrition in Metabolic Diseases Kashan University of Medical Sciences Kashan Iran

**Keywords:** Condition place preference, Dopamine, Morphine, Rat, Vitamin D

## Abstract

**Introduction:**

Despite half a century of research on vitamin D (Vit. D), its link to substance abuse and dependence has only been discussed in recent decades. Evidence also shows the involvement of Vit. D in the evolution of dopaminergic neurons in the nucleus accumbens, an increase in the expression of tyrosine hydroxylase, and the regulation of dopaminergic processes. The novel idea for this work is taken from a hypothesis given about the effectiveness of Vit. D on dopamine signaling pathway. It is therefore presumed that Vit. D can be considered an effective therapeutic approach for narcotic addiction and substance abuse.

**Methods:**

The animals were assigned into six groups (control, vehicle, Morphine [Mor.], and Vit. D [250, 500, and 1000 IU/kg, i.p.]). Following each conditioning session in a conditioned place preference (CPP) model, the animals received Vit. D. Afterward, the locomotor activity of the animals was assessed using open‐field apparatus. Malondialdehyde (MDA), nitric oxide (NO), catalase (CAT), superoxide dismutase (SOD), thiol, and total antioxidant capacity (TAC) were measured in the brain. The relative DRD2 and GDNF expressions (%) were also measured in the hippocampus.

**Results:**

Vit. D administration after Mor. caused a significant increase in the place preference index in the acquisition phase (*p* < .05). Vit. D altered the oxidation/antioxidation profiles (CAT, SOD, MDA, NO, TAC, and Thiol). Vit. D was more effective than Mor. in the expression of GDNF (*p* < .0001); however, in the expression of DRD2, this was only the case for 1000 IU Vit. D (*p* < .0001).

**Conclusions:**

Considering the increased place preference index induced by Mor., it can be concluded that Vit. D interacts via the oxidative pathway and DRD2–GDNF signaling to potentiate the Mor. effect.

## Graphical abstract

Vitamin D can be considered an effective therapeutic approach for narcotic addiction and substance abuse.

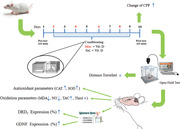

## 1 INTRODUCTION

Drug dependence is a disappointing concern in many communities that results in divesting physical and emotional problems. Despite applying noneffective routine approaches, there have been higher rates of relapse among recovered cases (Moeini et al., 2019). After periods of abstinence, the relapse rate is high among detoxified opioid addicts and this tendency is one of the main clinical problems in the treatment of addiction (Wise, 2002).

Vitamin D (Vit. D) is a secosteroid hormone known for its classical role in bone metabolism and calcium uptake. Calcium is involved in bone health and has a role in many essential processes. Some nonclassical functions have been reported for Vit. D, including antioxidant effects, immunomodulation, modulation of epithelial–mesenchymal transition, antiapoptosis effects, and anti‐inflammatory activities (Banafshe et al., 2019; Hamden et al., 2009; Kesby et al., 2006; Sanchez et al., 2009; Szeto et al., 2007).

Over the last decade, experimental evidence has shown that Vit. D plays a critical role in brain function and development. Despite over 50 years of research on Vit. D, its connection with neuropsychiatric disorders (e.g., drug addiction) has only been suggested in the last two decades (Eyles et al., 2009). Evidence from the adult brain has confirmed the neuroprotective effects of Vit. D on the dopaminergic pathways. Administering 6‐hydroxydopamine, a selective dopaminergic toxin, in animal subjects pretreated with 1,25‐dihydroxyvitamin D3 for 1 week has helped preserve the dopaminergic function (Eyles et al., 2009). Rats postnatally treated with a single dose of Vit. D have shown higher dopamine (DA) in the brainstem and changes in the hypothalamus and caudate putamen (Tekes et al., 2009). The in vitro administration of 1,25‐dihydroxyvitamin D3 in adrenal medullary cells resulted in the overexpression of tyrosine hydroxylase (Kesby et al., 2011). An increasing body of evidence implies that Vit. D can regulate dopaminergic processes (Cass et al., 2012; Puchacz et al., 1996; Sanchez et al., 2009).

Opioids and substance abuse may enhance oxidative stress (OS) factors (Salarian et al., 2018). Drug abuse also leads to mineral loss and nutritional deficiency, similar to Vit. D and protein deficiency, which can cause susceptibility to caries and negatively affect the tooth structure (Eserian, 2013; Sebastiani et al., 2018). Earlier documents have demonstrated low bone mineral density and hypovitaminosis D in opioid‐dependent patients under methadone maintenance (Kim et al., 2009, 2006). An entirely new study published by Kemeny et al. (2021) reported that Vit. D insufficiency aggravates opioid addiction and makes lab animals sensitive to morphine (Mor.) reward (Kemény et al., 2021). A previous research on human models revealed the association of Vit. D deficiency with greater opioid dosages among chronic opioid consumers (Turner et al., 2008). Another study on the subject showed the possible contribution of Vit. D to neurodevelopment, which may exert a neuroprotective impact on the dopaminergic pathways in adults’ brains (Eserian, 2013). Cass et al. (2006) showed that the oral administration of Vit. D may decrease metabolites and protect the dopaminergic system against the depletion of serotonin and DA in Vit. D‐treated animals that were given methamphetamine. Studies have also established the right duration of treatment with Vit. D, and 8 days of Vit. D consumption (1.0 g/kg/day) upregulated GDNF expression and the level of proteins in the brain (Sanchez et al., 2009). The development of dopaminergic neurons in the ventral tegmental area (VTA) may be affected by the modified GDNF, which causes changes in DA release in the “nucleus accumbens.” Furthermore, hypovitaminosis D‐induced reductions in GDNF can modify the regular development of the dopaminergic pathways (Kesby et al., 2006). Accordingly, OS, inflammatory parameters, and the metabolic profile can be influenced by Vit. D through the upregulation of antioxidant systems and the reduced activation of the pro‐inflammatory transcription factor nuclear factor‐kappa B (Hamden et al., 2009; Szeto et al., 2007).

Conditioned place preference (CPP) is a behavioral paradigm that allows researchers to observe the effects of drugs of abuse on associative learning in a context‐dependent manner (Wainwright et al., 1998). Contrary to instrumental learning paradigms, in which reward is subject to response, CPP is a classical conditioning paradigm that enables associating neutral stimuli with reward in order to elicit appropriate responses (Kemény et al., 2021). Based on various studies, CPP is very effective in determining the positively reinforcing effects of morphine (Wainwright et al., 1998).

Considering the aforementioned findings and the hypothesis first proposed by Jaqueline Kalleian Eserian on this regard (Eserian, 2013), this study was conducted to ascertain whether Vit. D can reduce Mor. tendency in rats through OS markers using a CPP paradigm. The study will also investigate the effects of Vit. D on the D2 dopamine receptor (DRD2) and GDNF expression in rats.

## 2 METHODS

### 2.1 Animals

All the experiments were carried out on male Wistar rats procured from the Kashan University of Medical Sciences (KAUMS), weighing 200–220 g at the beginning of the experiment. The animals were kept in an animal house in a normal 12‐h light/12‐h dark cycle with free access to food and water at all times. The laboratory temperature was controlled at 22–25°C. All the behavioral tests were carried out between 8:00 a.m. and 2:00 p.m. All the experimental procedures were performed according to the ethical guidelines of KAUMS, and the study was registered and approved by the Ethics Committee of the university (code: IR.KAUMS.MEDNT.REC.1397.090).

### 2.2 Experimental design

Thirty‐six rats were randomly assigned into six groups: control (Ctl.), Mor., vehicle (Veh.), and three treatment groups (*n* = 6 per group). Group I (Ctl.) received saline following the conditioning phase for eight consecutive days. Group II (Mor.) was treated with alternate injections of either Mor. (days 2, 4, 6, and 8) or saline (days 3, 5, 7, and 9) following the conditioning phase for eight consecutive days. Group III (Veh.) received Mor. (days 2, 4, 6, and 8) or saline (days 3, 5, 7, and 9) following the conditioning phase and was given almond oil, immediately after the conditioning phase for eight consecutive days. Groups IV–VI (treatment groups) received Mor. (days 2, 4, 6, and 8) or saline (days 3, 5, 7, and 9) following each conditioning session and were given Vit. D (250, 500, and 1000 IU/kg, i.p.) immediately after every conditioning session for eight consecutive days. Based on the hypothesis by Jaqueline Kalleian Eserian (see Section 1) that declared “The conditioning regime should be 8 days long, and Vit. D should be given immediately at the end of each conditioning session,” we designed our experiments. On the completion of the CPP tests, the locomotor activity of the animals was measured using an open‐field apparatus. After the termination of the behavioral experiments, all the animals in the six groups were sacrificed and their extracted brain regions (the cortex and hippocampus) were stored at −70°C until further processing for biochemical estimations and molecular quantification.

### 2.3 Drugs and treatments

Mor. sulfate was purchased from Temad Co. (Tehran, Iran). The preliminary dose–response was selected based on our pilot study. A fresh Mor. solution was prepared for subcutaneous injection by dissolving in sterile saline 0.9%. Since the 5 mg/kg dose of Mor. effectively induced CPP, in contrast to the other doses (2.5, 5, 7.5, and 10 mg/kg), it was chosen as the effective dose. Vit. D (Daroupakhsh Pharmaceutical Co., Tehran, Iran) was diluted in almond oil and prepared to the selected doses freshly. The Vit. D doses (250, 500, and 1000 IU/kg, i.p.) were selected based on our previous study (Banafshe et al., 2019) and were injected on the second to ninth days after each conditioning session.

### 2.4 Behavioral testing

#### 2.4.1 Conditioned place preference

The CPP apparatus (Maze Router, Tabriz, Iran) contains three chambers (called A, B, and C) that are separated by guillotine doors. Two large conditioning chambers (chambers A and B) are in the same size but in different colors and patterns. Chamber A has walls and a floor, which are black and white in pattern, while the walls and floor of chamber B are white. Chamber C is located in the middle of chambers A and B and is smaller than them, and a guillotine door connects chambers A and B (Ghavipanjeh et al., 2015).

The CPP protocol was followed continuously for 10 days and consisted of three distinct phases (Thorn et al., 2012): preconditioning, conditioning, and postconditioning tests. A week before beginning the experiments, all the animals were allowed to become habituated to the apparatus. After their habituation, in the preconditioning phase (on day 1), the animals were given a test in which they were placed in the middle chamber for 15 min while the guillotine doors were raised so as to allow access to the entire apparatus. The time spent in each chamber was recorded for this phase. During the conditioning phase (on days 2–9), each rat was treated with alternate injections of either Mor (5 mg/kg, i.p.) or saline (2 ml/kg, i.p.) for 8 days; the rats were thus treated with Mor on days 2, 4, 6, and 8 and with saline on days 3, 5, 7, and 9. The rats were confined to the Mor. compartment for 45 min immediately after Mor. administration and to the saline compartment immediately after saline injection through an “unbiased” procedure. In the postconditioning trial (on day 10), a CPP test was given. In this phase, the animals were placed in the middle chamber while the guillotine doors were removed, and similar to day 1, they were allowed free access to the entire apparatus for 15 min. The time spent in each chamber was also recorded for this phase. The change of preference was calculated as the difference (in second) between the times spent in the Mor‐receiving chamber on day 10 and on day 1 (Chen et al., 2019; Ghavipanjeh et al., 2015).

#### 2.4.2 Open‐field test

Following the completion of the CPP tests on day 12, all the rats were tested in an open‐field apparatus to measure their locomotor activity, as reported (Alinaghipour et al., 2019). Locomotor activity was evaluated by placing the rat inside a black, metal open‐field box (40 × 40 × 30 cm) and leaving it to explore the box for 5 min. Activity in the open field was quantified by a computer‐based CCD camera and a digital optical animal activity system (MazeRouter, Tabriz, Iran). The total distance traveled (cm) was recorded as the measure of locomotor activity.

### 2.5 Oxidation profiles

Malondialdehyde (MDA) levels were measured according to the method proposed by Ohkawa et al. (1979) to determine lipid peroxidation (LPO) in the brain tissue in terms of nmol/mg protein (Ardjmand et al., 2019).

### 2.6 Antioxidant profiles

The activities of catalase (CAT), superoxide dismutase (SOD), total antioxidant capacity (TAC), and thiol were measured by ELISA according to the manufacturer's instructions (Kiazist Co., Iran). The nitric oxide (NO) levels were determined in the brain tissue in nmol/mg protein by measuring the supernatant metabolites using Griess reagent as per the method proposed by Moshage et al. (1995).

### 2.7 Total protein

Total protein levels were measured in the brain tissue using the Bradford method with concentrated Coomassie blue reagent and bovine serum albumin as the standards (Hammond & Kruger, 1988).

### 2.8 Western blotting

Total proteins were extracted from each group using RIPA lysis buffer containing 50 mM Tris (pH 8), 150 mM NaCl, 0.5% sodium deoxycholate, 0.1% sodium dodecyl sulphate (SDS), and Triton X‐100 0.1% and protease inhibitor cocktail (Melford). The total protein concentration was assayed according to Bradford's method using bovine serum albumin protein as a reference standard. The extracted proteins, equivalent to 50 μg of proteins, were electrophoresed on SDS–polyacrylamide gel 12% and then transferred to polyvinylidene fluoride membranes. The membranes were blocked in nonfat dry milk‐TBST 5% (20 mM Tris‐HCl [pH 7.6], Tween 20 0.1%, 150 mM NaCl), probed with specific antibodies (1/4000) at 4°C overnight. The blots were washed twice with TBST and then incubated for 2 h at room temperature with rabbit IgG conjugated to horse HRP (1/10,000) as the secondary antibody. Immunoreactive polypeptides were detected by Amersham ECL Select Western Blotting Detection Reagent (Amersham Bioscience, USA) and subsequent autoradiography. To normalize the loaded protein, the blots were stripped in stripping buffer containing 100 mM 2‐mercaptoethanol, 2% (w/v) SDS, and 62.5 mM Tris–HCl (pH 6.8) and then probed with anti‐β‐actin antibody (1/4000). The quantification of the integrated densities of the bands was performed by Image J software (Alinaghipour et al., 2019).

### 2.9 Statistical analysis

The results are presented as mean ± SEM. Data were analyzed using the one‐way ANOVA and Tukey's post hoc test. The calculations were performed in Prism software (version 9). A *p*‐value of <.05 was taken as the level of statistical significance.

## 3 RESULTS

### 3.1 Behavioral tests following Mor. and Mor. + Vit. D in CPP and open field

**FIGURE 1:**
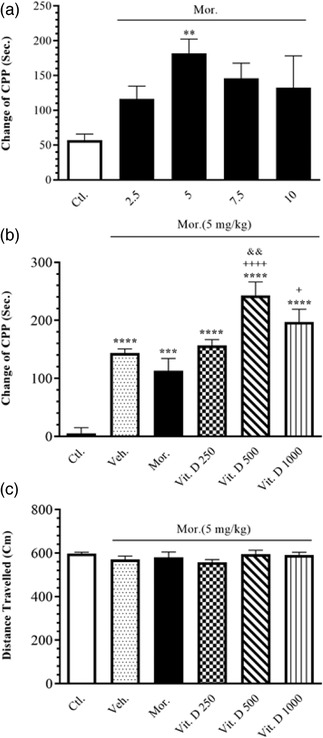
(a) The effects of different doses of Mor. administration on CPP for determining the effective dose of Mor. (b) The effect of treatment with different dose of Vit. D (250, 500, and 1000 IU/kg, i.p.) on the CPP in all groups. (c) The effect of treatment with different dose of Vit. D (250, 500, and 1000 IU/kg, i.p.) on locomotor activity in all groups. Ctl., Control; Veh., vehicle; Mor., Morphine; Vit. D, vitamin D; CPP, conditioned place preference. Data are expressed as mean ± SEM (*n* = 6 in each group). ^**^ *p* < .01 compared to Ctl. group. ^***^ *p* < .001 compared to Ctl. group. ^+^ *p* < .05 compared to Mor. group. ^+++^ *p* < .001 compared to Mor. group. ^&&^ *p* < .01 compared to Veh. group. Comparisons between different groups were made using one‐way analysis of variance (ANOVA) followed by Tukey's post hoc test

The effect of different doses of Mor. (2.5, 5, 7.5, and 10 mg/kg) on CPP was calculated in all the groups. The one‐way analysis of variance (ANOVA) revealed a significant increase in CPP change only with the 5 mg/kg dose compared to the saline group (*F*_4, 23_ = 3.71, *p* = .01). Therefore, 5 mg/kg of Mor. was taken as the effective dose in this study (Figure 1a).

Figure 1b shows the effects of treatment with an effective dose of Mor. alone (5 mg/kg) or its combination with different doses of Vit. D (250, 500, and 1000 IU/kg, i.p.) on CPP changes in all the groups. The one‐way ANOVA showed a significant difference between the groups (*F*_5, 39_ = 21, *p* = .0001). In addition, Tukey's post hoc test also showed an increase in CPP changes with 500 IU (*p* < .0001) and 1000 IU (*p* < .05) of Vit. D compared to the Mor. group. Moreover, the post hoc Tukey's test also showed an increase in CPP changes with 500 IU (*p* < .01) of Vit. D compared to the Veh. group.

The data also showed that treatment with different doses of Vit. D had no significant effects on locomotor activity in the open‐field apparatus (*F*_5, 36_ = 0.8839, *p* = .5) (Figure 1c).

### 3.2 The antioxidant parameters in the hippocampus following Mor. + Vit. D

**FIGURE 2:**
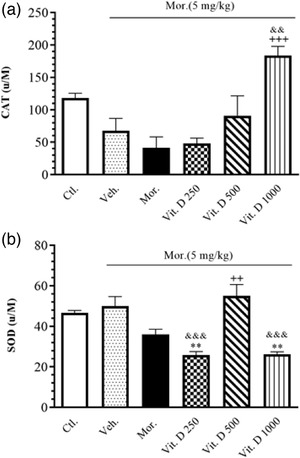
The activity of CAT (μM/mg protein) (a) and SOD (U/M) (b) in brain tissue after Vit. D treatment in all groups. Ctl., Control; Veh., vehicle; Mor., Morphine; Vit. D, vitamin D; CAT, catalase; SOD, superoxide dismutase. Data are expressed as mean ± SEM (*n* = 6 in each group). ^+++^ *p* < .001 compared to Mor. group. ^**^ *p* < .01 compared to Ctl. group. ^&&^ *p* < .01 and ^&&&^ *p* < .001 compared to Veh. group. Comparisons between different groups were made using one‐way analysis of variance (ANOVA) followed by Tukey's post hoc test

Figure 2 presents the effect of treatment with different doses of Vit. D (250, 500, and 1000 IU/kg, i.p.) on the tissue‐level activities of CAT and SOD in all the groups. The one‐way ANOVA indicated a significant increase in the antioxidant activities of CAT (*F*_5, 21_ = 8.656, *p* = .0001) in the Vit. D 1000 IU/kg group compared to the Mor. group. Moreover, the post hoc Tukey's test also showed a significant increase in the antioxidant activities of CAT (*F*_5, 21_ = 8.656, *p* = .001) in the Vit. D 1000 IU/kg group compared to the Veh. group (Figure 2a).

Also, a significant increase was observed in the antioxidant activity of SOD (*F*_5, 15_ = 9.63, *p* < .0001) in the Vit. D 500 IU/kg group compared to the Mor. group. Moreover, the post hoc Tukey's test also showed a significant decrease in the antioxidant activities of SOD with 250 IU (*p* < .001) and 1000 IU (*p* < .001) of Vit. D compared to the Veh. group (Figure 2b).

### 3.3 The oxidation parameters in the hippocampus following Mor. + Vit. D

**FIGURE 3:**
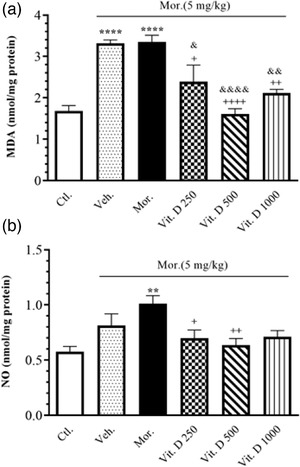
The level of MDA (nmol/mg protein) (a) and NO (nmol/mg protein) (b) in brain tissue after Vit. D treatment in all groups. Ctl., Control; Veh., vehicle; Mor., Morphine; Vit. D, vitamin D; MDA, malondialdehyde; NO, nitric oxide. Data are expressed as mean ± SEM (*n* = 6 in each group). ^**^ *p* < .01 compared to Ctl. group. ^***^ *p* < .001 compared to Ctl. group. ^+^ *p* < .05 compared to Mor. group. ^++^ *p* < .01 compared to Mor. group. ^+++^ *p* < .001 compared to Mor. group. ^&^ *p* < .05 compared to Veh. group. ^&&^ *p* < .01 and ^&&&&^ *p* < .0001 compared to Veh. group. Comparisons between different groups were made using one‐way analysis of variance (ANOVA) followed by Tukey's post hoc test

Figure 3a shows the effect of treatment with different doses of Vit. D (250, 500, and 1000 IU/kg, i.p.) on LPO in all the treatment groups. A significant increase was observed in MDA levels in the brain tissue in the Mor. and Veh. groups compared to Ctl. (*F*_5, 24_ = 14.88, *p* < .0001). A significant decrease in MDA levels in the brain tissue was also observed in the Vit. D 250 (*p* < .05), Vit. D 500 (*p* < .001), and Vit. D 1000 (*p* < .01) groups compared to Mor. Moreover, the post hoc Tukey's test also showed a significant decrease in MDA levels in the brain tissue in the Vit. D 250 (*p* < .05), Vit. D 500 (*p* < .001), and Vit. D 1000 (*p* < .01) groups compared to Veh.

Figure 3b shows the effect of treatment with different doses of Vit. D (250, 500, and 1000 IU/kg, i.p.) on NO in all the experimental groups. A significant increase was observed in NO levels in the brain tissue in the Mor. group compared to the Ctl. (*F*_5, 23_ = 4.941, *p* = .003). A significant decrease was also observed in NO levels in the brain tissue in the Vit. D 250 (*p* < .05) and Vit. D 500 (*p* < .01) groups compared to the Mor. group.

**FIGURE 4:**
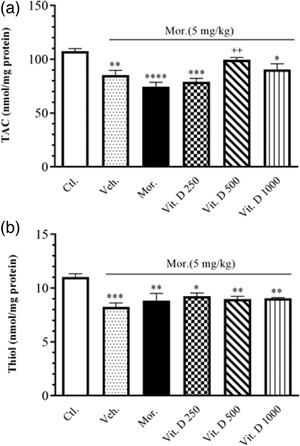
The level of TAC (nmol/mg protein) (a) and the level of Thiol (nmol/mg protein) (b) in brain tissue after Vit. D treatment in all groups (*n* = 10 in each group). Ctl., Control; Veh., vehicle; Mor., Morphine; Vit. D, vitamin D; TAC, total antioxidant capacity. Data are expressed as mean ± SEM (*n* = 6 in each group). ^*^ *p* < .05 compared to Ctl. group. ^**^ *p* < .01 compared to Ctl. group. ^***^ *p* < .001 indicates significant difference compared to Ctl. group. ^++^ *p* < .01 compared to Mor. group. Comparisons between different groups were made using one‐way analysis of variance (ANOVA) followed by Tukey's post hoc test

Figure 4a shows the effect of treatment with different doses of Vit. D (250, 500, and 1000 IU/kg, i.p.) on TAC in all the treatment groups. A significant decrease was observed in TAC levels in the brain tissue in the Mor. group compared to Ctl. (*F*_5, 24_ = 10.78, *p* = .0001). A significant increase was observed in TAC levels in the brain tissue in the Vit. D 500 group (*p* < .01) compared to Mor.

Figure 4b shows the effect of treatment with different doses of Vit. D (250, 500, and 1000 IU/kg, i.p.) on thiol in all the treatment groups. A significant decrease was observed in thiol levels in the brain tissue in the Mor. group compared to Ctl. (*F*_5, 22_ = 7.664, *p* = .0003). No significant change was discerned in thiol levels in the brain tissue in the other groups compared to Mor.

### 3.4 The hippocampal DRD2/β‐Actin expression levels following Mor. + Vit. D

**FIGURE 5:**
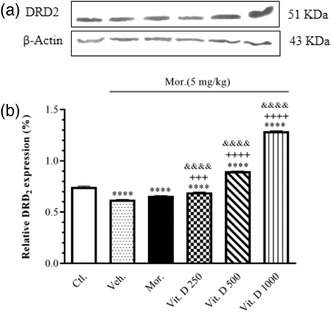
The effect of Mor. alone and Mor. + Vit. D (250, 500, and 1000 IU/kg, i.p.) on DRD2 expression (%). All animals received saline (1 ml/kg) or effective dose of Mor. (5 mg/kg, i.p.) per se or Mor. and different doses of Vit. D (250, 500, and 1000 IU/kg, i.p.). (a) The brain expression levels of DRD2, in Mor.‐induced CPP. The evaluation of DRD2 expression level was performed in six groups: Ctl. (group 1, no treatment and CPP); Veh. (group 2); (Mor. + almond oil) the Mor. (5 mg/kg) (groups 3); and Mor. + Vit. D groups (groups 4, 5, and 6) following CPP using western blotting (upper panel shows the immunoblotting profile). (b) The brain expression levels of DRD2, in CPP paradigm. The analysis indicated that DRD2 was significantly decreased (*p* < .001) in Veh. group and increased in Mor. + Vit. D groups as compared to Ctl. group. ^****^ *p* < .0001 compared to the Ctl. group, ^+++^ *p* < .001 and ^++++^ *p* < .0001 compared to the Mor. group. ^&&&&^ *p* < .0001 compared to Veh. group. Each value represents the mean ± SEM (*n* = 6 in each group). Comparisons between different groups were made using one‐way analysis of variance (ANOVA) followed by Tukey's post hoc test. Ctl., Control; Veh., vehicle; Mor., Morphine; Vit. D, vitamin D

Figure 5a shows the relative DRD2 expression (%) in the hippocampus for the administration of Mor. + Vit. D in the CPP via western blot analysis. The densitometric analysis revealed significant dose‐related changes in hippocampal DRD2 expression.

Figure 5b reveals the overexpression of the DRD2 in the rats’ hippocampus in the Mor. + Vit. D‐administered groups in the CPP model compared with the Ctl. group receiving saline (*F*_5, 12_ = 5537, *p* = .0001) (*p* < .001 for all the groups). The results also showed a significant increase in the overexpression of the DRD2 in the rats’ hippocampus in the Vit. D 250 (*p* < .001), Vit. D 500 (*p* < .0001), and Vit. D 1000 (*p* < .0001) groups compared to Mor. Moreover, the post hoc Tukey's test also showed a significant increase in the overexpression of the DRD2 in the rats’ hippocampus in the Vit. D 250 (*p* < .0001), Vit. D 500 (*p* < .0001), and Vit. D 1000 (*p* < .0001) groups compared to Veh.

### 3.5 The hippocampal GDNF/β‐Actin expression levels following Mor. + Vit. D in the CPP

**FIGURE 6:**
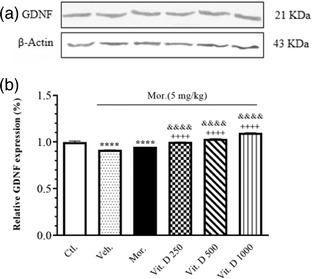
The effect of Mor. alone and Mor. + Vit. D (250, 500, and 1000 IU/kg, i.p.) on GDNF expression (%). All animals received saline (1 ml/kg) or effective dose of Mor. (5 mg/kg, i.p.) per se or different doses of Vit. D (250, 500, and 1000 IU/kg, i.p.). (a) The brain expression levels of GDNF, in Mor.‐induced CPP. The evaluation of GDNF expression level was performed in six groups Ctl. (group 1, no treatment and CPP); Veh. (group 2); (Mor. + almond oil) the Mor. (5 mg/kg) (groups 3); and Mor. + Vit. D groups (groups 4, 5, and 6) following CPP using western blotting (upper panel shows the immunoblotting profile). (b) The brain expression levels of GDNF, in CPP paradigm. The analysis indicated that GDNF was significantly decreased (*p* < .001) in Veh. group and increased in Mor. + Vit. D groups as compared to Ctl. group. ^****^ *p* < .001 compared to the Ctl. group. ^++++^ *p* < .001 compared to the Mor. group. ^&&^ *p* < .01 and ^&&&&^ *p* < .0001 compared to Veh. group. Each value represents the mean ± SEM (*n* = 6 in each group). Comparisons between different groups were made using one‐way analysis of variance (ANOVA) followed by Tukey's post hoc test. Ctl., Control; Veh., vehicle; Mor., Morphine; Vit. D, vitamin D

Figure 6a illustrates the relative GDNF expression (%) in the hippocampus with the administration of Mor. + Vit. D in the CPP via western blot analysis. The densitometric analysis revealed significant dose‐related changes in the hippocampus expression of GDNF.

Figure 6b shows the overexpression of GDNF in the rats’ hippocampus in the Mor. + Vit. D‐administered groups in the CPP model compared to the Ctl. group receiving saline (*F*_5, 12_ = 230.9, *p* = .0001). The results also showed a significant increase in the overexpression of the GDNF in the rats’ hippocampus in the Vit. D 250 (*p* < .0001), Vit. D 500 (*p* < .0001), and Vit. D 1000 (*p* < .0001) groups compared to Mor. Moreover, the post hoc Tukey's test also showed a significant increase in the overexpression of the GDNF in the rats’ hippocampus in the Vit. D 250 (*p* < .0001), Vit. D 500 (*p* < .0001), and Vit. D 1000 (*p* < .0001) groups compared to Veh.

## 4 DISCUSSION

According to the present findings, 5 mg/kg of Mor. resulted in Mor‐induced CPP. A combination of this dosage of Mor. with Vit. D (500–1000 IU) augmented the CPP response. Interestingly, contrary to our expectation, both Veh and Mor. (5 mg/kg) have shown similar changes in CPP. This apparent contradiction could be due to the fact that the general condition of both groups (except for taking Veh.) was the same. Overall, no significant difference was observed in locomotor activity in the open‐field test in any of the groups.

As suggested by Eserian (2013), whose novel hypothesis triggered the design of the present study, one possible way to verify his innovational hypothesis is to use Vit. D in animals by a CPP model (Nestler, 1994). CPP is a well‐known model commonly used to evaluate the mechanism of drug abuse (Wainwright et al., 1998). The CPP paradigm affords both spatial/visual and tactile cues using different patterns and textures on walls and the floor using a random unbiased pairing method (Eserian et al., 2012). Based on the results reported by Sanchez et al. (2009), an 8‐day CPP protocol was used in the present study, since it had successfully upregulated GDNF expression and protein levels in the brain instead of the 4‐day protocol with the same Vit. D dose recently reported by Kemény et al. (2021). Their study proved the theory that impaired Vit. D receptor signaling makes animals susceptible to opioid‐seeking behavior, which is reversible by Vit. D administration (Kemény et al., 2021).

The measurement of antioxidant parameters showed a significant increase in CAT levels in the Mor. + Vit. D 1000 IU/mg/kg administration group; however, the Mor. + Vit. D 500 IU/mg/kg group had a significant increase in SOD compared to the Mor. group.

The measurement of oxidation parameters following the administrations of all the doses of Vit. D (especially 500 IU) decreased MDA levels significantly. Vit. D at doses of 250 and 500 IU (especially 500 IU) reduced NO levels significantly. Nevertheless, only 500 IU of Vit. D increased TAC. Meanwhile, none of the Vit. D doses had a significant effect on thiol levels.

Brain is highly susceptible to peroxidative damage, since it contains a relatively high degree of unsaturated fatty acids in relation to its level of antioxidants and consumes more oxygen. Mor. can induce oxidative cell injury and neurodegeneration in neuronal cells. In addition, Mor can be metabolized into free radicals, and the overproduction of reactive oxygen species (ROS) can lead to oxidative damage (Sumathi et al., 2011), which should be considered in the treatment of opioid dependence (Salarian et al., 2018). In the present study, brain concentrations of MDA and NO were higher in the Mor. group compared to Ctl. One can assume that increased MDA and NO suggest the inability of Mor. to protect the brain from OS. Nevertheless, all the administered doses of Vit. D (especially 500 IU) decreased MDA and NO levels significantly. In line with the present findings, these results further support the antioxidant effect of Vit. D and potential tissue protection in lipid oxidation and inflammation damages (Mathieu et al., 2005). Vit. D has also been reported to improve glutathione levels and prevent inducible NO synthase (iNOS) generation (Cass et al., 2006). Other studies have reported that Vit. D has protective effects in human retinal cells by improving the antioxidant defense; in addition, oral Vit. D administration along with calcium resulted in higher SOD activity, CAT, and glutathione peroxidase in diabetic rats (Fernandez‐Robredo et al., 2020).

The present study further showed that all the administered doses of Vit. D (especially 500 IU) significantly increased SOD activity and increased CAT activity (especially 1000 IU) compared to the Mor. group. Nevertheless, only the 500‐IU dose of Vit. D increased TAC levels.

Furthermore, the finding that Vit. D improved glutathione levels and prevented iNOS generation, in turn, could decline methamphetamine toxicity to the DA system by decreasing methamphetamine free radicals’ generation (Cass et al., 2006). Likewise, the Vit. D‐treated animals exhibited a remarkable attenuated methamphetamine‐mediated decline in DA and metabolites comparing to controls. It was suggested that Vit. D protects the dopaminergic system against the reducing effects of methamphetamine (Cass et al., 2006). In another study by Fernandez‐Robredo et al. (2020), Vit. D had protective effects in human retinal cells by improving the antioxidant defense. A study on diabetic rats found that oral Vit. D intake with calcium resulted in higher SOD activity, CAT, GPX, and a lower level of MDA compared to controls (Alatawi et al., 2018). Collectively, these results further support the antioxidant effect of Vit. D and its potential tissue protection effect against lipid oxidation and inflammation damage (Mathieu et al., 2005). Another study also showed that long‐term Vit. D deficiency elevates ROS in the brain (Keeney et al., 2013) and treatment with Vit. D reduces ROS production, NO accumulation, and iNOS expression in cerebral neurons in isolated tissues (Huang et al., 2015).

Nevertheless, the repeated administration of opioids such as Mor led to the production of ROS, weakened the antioxidant capacity, regulated synaptic plasticity, and finally contributed to opiate dependence (Sumathi et al., 2011). Similarly, synaptosomal evidence from Vit. D‐deficient animals revealed that higher ROS production was reversed in rats by Vit. D supplementation (Kasatkina et al., 2020). Also, the established neuroprotective mechanism of Vit. D showed the blockade of NO (Emmanuel Garcion et al., 2002) and iNOS in the animal brain (Garcion et al., 1997, 1998).

We measured GDNF and DRD2 expression in the hippocampus, as an important structure in addiction/reward circuit (Khalil‐Khalili et al., 2018). According to our molecular experiments, the effect of Vit. D (in all doses) was more potent than Mor. with respect to GDNF expression; however, only 1000 IU of Vit. D had a significant impact compared to Mor. on DR2D expression. Consistent with this finding, the treatment of neural stem cells with Vit. D revealed the overexpression of NT‐3, BDNF, and GDNF (Shirazi et al., 2015), and in vitro neurons showed GDNF expression and DA cell proliferation following Vit. D treatment, which could be reversed with GDNF antagonism (Orme et al., 2013).

The DA system genes (e.g., rs1076560 and D2 [DRD2]) are potential candidates to carry over the risk variants because the rewarding effects of drugs of abuse are mediated by DA neurotransmission (Clarke et al., 2014).

Several new studies have found connections between Vit. D levels and its receptor activity with various health conditions, including chronic pain development in drug addiction, which is a leading cause of disease burden and disability (Habib et al., 2020). Previous studies have demonstrated the important role of D2 receptors in opioid dependence. Opioid dependence is associated with low striatal DA D2 receptor binding and DA release (Martinez et al., 2012). A link has also been reported between the D2 receptor and heroin dependence. DRD2 TaqI A1 allele carriers have also been shown to be prone to heroin abuse (Hou & Li, 2009).

Vit. D induces endogenous GDNF expression significantly. It supports that the survival of dopaminergic neurons is the most critical feature of GDNF. GDNF is essential in the dopaminergic pathways and modulates dopaminergic cell apoptosis in the substantia nigra postnatally. It has been suggested that GDNF expression is reduced in the cerebrum of developmental Vit. D‐deficient neonatal rats. Dopaminergic neuron development within the VTA can be influenced by modified GDNF, resulting in DA release changes within the nucleus accumbens. Hypovitaminosis D‐induced decline in GDNF can apparently modify the well‐ordered development of the dopaminergic pathways (Eserian, 2013; Kesby et al., 2006). The protective effects of Vit. D are reportedly due to GDNF overexpression (Cass et al., 2006), as the direct administration of GDNF into the striatum before methamphetamine treatment has led to complete protection against methamphetamine's dopaminergic toxicity, such as reduced content and release of striatal DA (Cass, 1996).

To mention the limitations, the results of our study would be more interesting if we had measured the plasma levels of Vit. D and consequently had studied its augmenting effect on Mor., CPP, and so the Vit. D treatment. Another limitation is that our method for assaying the total NO was an indirect method. Hence, we recommend designing similar studies in future to use some direct and precise methods for NO assaying (e.g., electron paramagnetic resonance spectroscopy) (Kozlov et al., 1995).

To conclude, considering the increased place preference index induced by Mor., it can be concluded that Vit. D interacts via the oxidative pathway and DRD2–GDNF signaling to potentiate the Mor. effect. The biochemical findings of this study suggest the beneficial effects of Vit. D on antioxidative/oxidative profiles, GDNF, and DR2D expression. Taken together, these results demonstrate that Vit. D can be clinically evaluated as a treatment option for drug addiction. Nevertheless, further animal and human studies are recommended to confirm these findings.

## CONFLICT OF INTEREST

The authors declare no conflict of interest.

### PEER REVIEW

The peer review history for this article is available at: https://publons.com/publon/10.1002/brb3.2877

## Data Availability

The data that support the findings of this study are available from the corresponding author upon reasonable request.
